# Chromosome Segregation Proteins as Coordinators of Cell Cycle in Response to Environmental Conditions

**DOI:** 10.3389/fmicb.2020.00588

**Published:** 2020-04-15

**Authors:** Monika Pióro, Dagmara Jakimowicz

**Affiliations:** Department of Molecular Microbiology, Faculty of Biotechnology, University of Wrocław, Wrocław, Poland

**Keywords:** chromosome segregation, ParA, ParB, segrosome, cell division, cell elongation

## Abstract

Chromosome segregation is a crucial stage of the cell cycle. In general, proteins involved in this process are DNA-binding proteins, and in most bacteria, ParA and ParB are the main players; however, some bacteria manage this process by employing other proteins, such as condensins. The dynamic interaction between ParA and ParB drives movement and exerts positioning of the chromosomal origin of replication (*oriC*) within the cell. In addition, both ParA and ParB were shown to interact with the other proteins, including those involved in cell division or cell elongation. The significance of these interactions for the progression of the cell cycle is currently under investigation. Remarkably, DNA binding by ParA and ParB as well as their interactions with protein partners conceivably may be modulated by intra- and extracellular conditions. This notion provokes the question of whether chromosome segregation can be regarded as a regulatory stage of the cell cycle. To address this question, we discuss how environmental conditions affect chromosome segregation and how segregation proteins influence other cell cycle processes.

## Introduction

Bacteria must adjust their cell cycle to their environmental conditions. Unfavorable conditions such as starvation, oxidative, or osmotic stress alter the energetic state of the cell and trigger the stress signaling molecules (sigma factors, response regulators, signaling nucleotides) (Hengge, 2009; Gottesman, 2019; Latoscha et al., 2019; McLean et al., 2019). As the result, cells modify transcription, increase the generation time, completely inhibit cell division or sometimes form spores or enter dormancy (Errington, 2003; Jones et al., 2013; Heinrich et al., 2015; Desai and Kenney, 2019). While the primary cell cycle checkpoints are the initiation of replication and onset of cell division, these processes must be tightly coordinated with chromosome segregation (for recent reviews, see: Dewachter et al., 2018; Marczynski et al., 2019; Reyes-Lamothe and Sherratt, 2019; Burby and Simmons, 2020). Thus, the chromosome segregation process may link critical stages of the cell cycle.

The role of segregation proteins is to control the positioning of chromosomal (or plasmid) DNA during cell division. Importantly, in most bacterial cells, chromosome segregation begins soon after the initiation of chromosome replication and must be completed before the termination of cell division (Dewachter et al., 2018; Reyes-Lamothe and Sherratt, 2019). During chromosome replication, segregation proteins position newly duplicated chromosomal origin of replication (*oriC*) regions and ensure proper chromosome organization. Interestingly, in a number of bacterial species, positioning of the *oriC* and the pattern of chromosome organization may be modified in response to altered environmental conditions, such as limited nutrients (Wang et al., 2013; Badrinarayanan et al., 2015). Perfect examples of this modification are the profound changes in chromosome compaction observed during starvation-induced sporulation of *Bacillus subtilis* and *Streptomyces* spp. (Errington, 2001; Flärdh and Buttner, 2009; Jakimowicz and van Wezel, 2012). While in vegetatively growing *B. subtilis*, the *oriC* is shifted away from the cell pole, during the formation of endospores, which begins with asymmetric cell division, the *oriC* is anchored at the poles (Wang et al., 2014). However, even in non-sporulating bacteria, chromosome organization patterns may be altered depending on culture conditions; for example, in *Escherichia coli*, the chromosome arrangement changes from one in which the *oriC* adopts a mid-cell position in fast-growing cultures to a longitudinal in slow-growing cells in minimal media (Kleckner et al., 2014; Badrinarayanan et al., 2015). Chromosome topology is controlled by a set of proteins, predominantly topoisomerases and nucleoid-associated proteins (NAPs), whose activities were shown to be influenced by environmental and physiological factors (temperature, pH, salt concentration) (Dorman and Dorman, 2016; Dame et al., 2019). However, the mechanisms by which chromosome arrangement and segregation are adjusted to physiological state of bacterial cell only begun to emerge.

In this review, we discuss how chromosome segregation may be influenced by environmental stress, particularly nutrients limitation, and induced by this factor change of physiological conditions. Furthermore, we explore how changes in segregation protein activity may allow cells to adjust to particular conditions. To address these issues, we focus on the interactions of segregation proteins with DNA and the crosstalk between segregation proteins and their partners.

## The Functions of Chromosome Segregation Proteins

**FIGURE 1:**
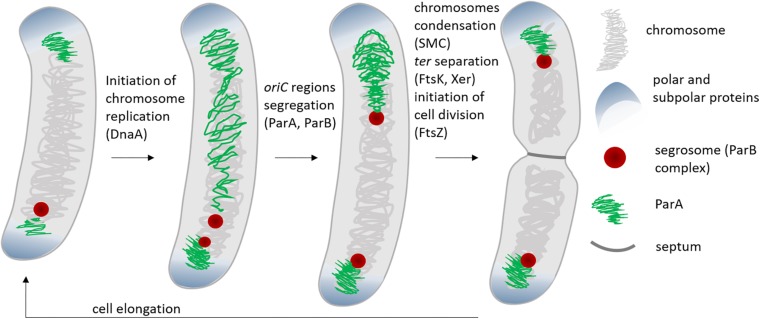
Stages of cell cycle and chromosome segregation in bacteria with polar or subpolar *oriC* localization (e.g., *C. crescentus*, *V. cholerae*, *M. smegmatis*, *M. xanthus*). The scheme shows the stage of the cell cycle when activity of DnaA – chromosome replication initiator, ParA, ParB, SMC, and FtsK – proteins involved in chromosome segregation, as well as FtsZ- cell division initiator is required.

Chromosome segregation in bacteria is governed by a set of proteins, among which ParA and ParB are key players. ParA and ParB were first identified as plasmid segregation proteins and further studies revealed that homologs of these proteins control positioning of the chromosomal *oriC* region (Lee et al., 2003; Mierzejewska and Jagura-Burdzy, 2012; Funnell, 2016). However, ParA and ParB are not fully widespread and chromosome segregation in some bacterial species (i.e., some γ-proteobacteria, including *E. coli*, which lack *parAB* genes) exploits the activities of other proteins, such as condensins (Nolivos and Sherratt, 2014; Dewachter et al., 2018). Condensins, which compact the bulk of chromosomal DNA, also play an auxiliary function in ParAB-dependent chromosome segregation (Graumann et al., 1998; Moriya et al., 1998; Wang et al., 2015). Segregation of the terminus-proximal region (*ter*) usually requires the activities of additional proteins, such as the DNA translocase FtsK or a type II topoisomerase specialized in DNA decatenation (TopoIV) (Kato et al., 1990; Yu et al., 1998; Dewachter et al., 2018) (Figure 1).

### The Role of ParA and ParB in *oriC* Segregation

The ParA and ParB proteins are components of the tripartite segregation system, which also involves *parS* sequences bound by ParB (*parABS* system) (Schumacher, 2008; Badrinarayanan et al., 2015). From 1 to 20 *parS* sequences, depending on the bacterial species, may be scattered over the *oriC*-proximal chromosomal region, which encompasses a range from 10 kb (in *Caulobacter crescentus*) to 200 kb in *Streptomyces coelicolor*, or even up to 650 kb in *Pseudomonas aeruginosa* (Jakimowicz et al., 2002; Livny et al., 2007; Tran et al., 2018; Kawalek et al., 2020). Upon interaction with ParB, the *parS* sequence-rich region engages in the formation of a large nucleoprotein complex named the segrosome (Funnell, 2014; Oliva, 2016). *parS* sites are often referred to as centromeric sites since they mark the chromosomal region that segregates first. Interestingly, in all studied bacterial species that use the ParABS system, ParB binding to DNA was reported to be maintained during majority of the cell cycle. In fact, the initiation of chromosome replication can be detected as the duplication of ParB complexes and the segrosomes mark the positions of the *oriC* regions throughout the whole cell cycle (Ptacin et al., 2010; Schofield et al., 2010; Shebelut et al., 2010; Harms et al., 2013; Trojanowski et al., 2015; Kois-Ostrowska et al., 2016) (Figure 1).

The organization of the ParB complex is still under investigation, but the studies to date have revealed that its architecture seems to be adjusted for the requirements of each particular bacterial species. Conserved structural features of ParB include the DNA-binding HTH motif in the central part of the polypeptide chain and two conserved sequences named ParB boxes in proximity to the N-terminus (Leonard et al., 2004; Schumacher and Funnell, 2005; Schumacher, 2007, 2008). While, the ParB boxes were shown to be required for segrosome formation, the N-terminal ParB domain is critical for interactions with ParA and C-terminal domain facilitates non-specific DNA binding (Autret et al., 2001; Schumacher and Funnell, 2005; Fisher et al., 2017). Non-specific interactions with DNA allow ParB to spread away from *parS* sites (Murray et al., 2006; Breier and Grossman, 2007; Kusiak et al., 2011; Chen et al., 2015; Taylor et al., 2015; Song et al., 2017). Moreover, ParB complex assembly was shown to require the bridging of protein molecules bound to spatially distant *parS* sites (Graham et al., 2014; Song et al., 2017; Soh et al., 2019). This bridging is mediated by dimerization of the arginine-rich ParB box II and was recently shown to be modulated by CTP binding in this region (Osorio-Valeriano et al., 2019; Soh et al., 2019). Notably, while in all bacterial species that use the *parABS* system, ParB specifically binds *parS* sequences, the affinity and specificity of ParB toward *parS* sequences vary among bacteria, resulting in differences in ParB spreading (Jalal et al., 2019). These differences, as well as variations in the number and distribution of *parS* sites, are reflected in the species-specific architecture of the ParB complex. Nevertheless, the primary role of the ParB complex is to organize the *oriC*-proximal region of the chromosome to facilitate its movement; the ParB complex thus performs a critical step in chromosome segregation.

The driving force for the chromosomal ParB complex is provided by a P-loop ATPase - ParA. Over the last decade, the hypothesized ParA mechanism of action has changed from filament formation to the generation of a dynamic concentration gradient (Ptacin et al., 2010; Lim et al., 2014; Vecchiarelli et al., 2014; Le Gall et al., 2016). Pivotal for the gradient-based model is non-specific DNA binding by ATP-bound ParA dimers (Leonard et al., 2005; Hester and Lutkenhaus, 2007). Interaction with segrosomes stimulates ParA ATPase activity, while ATP hydrolysis triggers dimer dissociation and protein release from DNA. This generates a ParA-depleted zone in proximity to the ParB complex. Due to their high affinity for DNA-bound ParA dimers, segrosomes move away from the depletion zone toward higher ParA concentration. The directionality of the ParA gradient and ParB movement was suggested to be enhanced by interactions between ParA and the polar or subpolar proteins: TipN and PopZ in *C. crescentus*, the bactofilin complex in *Myxococcus xanthus*, HubP in *Vibrio cholerae* and DivIVA in *Mycobacterium smegmatis* (Bowman et al., 2008; Ebersbach et al., 2008; Yamaichi et al., 2012; Ginda et al., 2013; Lin et al., 2017). These interactions are critical for proper *oriC* subcellular localization (Bowman et al., 2008; Ebersbach et al., 2008; Yamaichi et al., 2012; Lin et al., 2017; Pióro et al., 2019). Thus, highly genus- or species-specific interactions play a role in the spatial coordination of chromosome segregation with other cell cycle processes and presumably adjust the segregation machinery to the requirements of the life cycle of a particular bacterium.

### The Additional Roles of ParAB Proteins

In addition to their main function in moving the *oriC* region, in *B. subtilis, C. crescentus*, and *Streptococcus pneumoniae*, segrosomes were demonstrated to serve as the loading platform for the condensin complex, which is composed of SMC and the accessory proteins ScpA and ScpB (Sullivan et al., 2009; Wang et al., 2015; Tran et al., 2017). Large, rod-shaped, coiled-coil SMC proteins form dimers due to interactions within the hinge region and ATP-binding head domains. ATP hydrolysis and DNA binding induce conformational changes in the dimer that allow DNA loop extrusion, providing the basis for DNA compaction (Nolivos and Sherratt, 2014; Ganji et al., 2018; Baxter et al., 2019; Marko et al., 2019). Since binding and ATP hydrolysis are crucial for condensin activity, the efficiency of chromosome compaction induced by SMC proteins is presumably dependent on ATP levels in the cell. Importantly, SMC protein loading requires ParB bridging activity (Graham et al., 2014; Wilhelm et al., 2015). Upon loading in proximity to *oriC*, condensins spread along the chromosome, inducing its overall compaction and longitudinal arrangement.

Finally, an additional function of the ParB complex is its cooperation with proteins engaged in cell division regulation, such as MipZ in *C. crescentus* and *Rhodobacter sphaeroides* and PldP as well as FtsZ in *Corynebacterium glutamicum* (Donovan et al., 2010; Dubarry et al., 2019). Recent studies also shown that ParB also cooperate with NOC and both proteins are required to prevent cell division over nucleoid in *B. subtilis* (Hajduk et al., 2019) (Table 1).

**TABLE 1:** Interaction between proteins engaged in chromosome segregation and their protein partners.

Microorganism	Segregation protein	Polar or subpolar protein	Replication protein	Chromosome organization protein	Cell division protein	Other cell cycle-involved protein
*B. subtilis*	Soj		DnaA (Murray and Errington, 2008)			
	Spo0J			SMC (Gruber and Errington, 2009)		
*C. crescentus*	ParA	TipN, PopZ (Ptacin et al., 2010; Schofield et al., 2010)				
	ParB	PopZ (Bowman et al., 2008; Ebersbach et al., 2008)			MipZ (Thanbichler and Shapiro, 2006)	
*C. glutamicum*	ParB	DivIVA (Donovan et al., 2012)			FtsZ, PldP (Donovan et al., 2010)	
*M. smegmatis*	ParA	DivIVA (Ginda et al., 2013)				DNA glycosylase (Huang and He, 2012)
*M. xanthus*	ParA	Bactofilin-PadC (Lin et al., 2017)				
*R. sphaeroides*	ParB				MipZ (Dubarry et al., 2019)	
*S. coelicolor*	ParA	Scy (Ditkowski et al., 2013)				ParJ (Ditkowski et al., 2010)
	ParB			TopA (Szafran et al., 2013)		
*S. pneumoniae*	ParB			SMC (Minnen et al., 2011)	CpsD (Nourikyan et al., 2015)	
*V. cholerae*	ParAI	HubP (Yamaichi et al., 2012)				

The variety of roles played by segregation proteins in the cell cycles of various bacterial species manifests in the plethora of phenotypes resulting from *parAB* deletion. While *parAB* genes were demonstrated to be essential in *C. crescentus* and *M. xanthus*, in a number of other bacterial species, including *B. subtilis*, *P. aeruginosa*, *M. smegmatis*, and *C. glutamicum*, elimination of ParA or ParB leads to chromosome segregation aberrations and mispositioning of the *oriC* region, eventually resulting in the formation of from 1 to 30% anucleate cells (recently comprehensively reviewed by Kawalek et al., 2020). In some bacteria (*V. cholerae* and *B. subtilis*), *parB* (but not *parA*) deletion increases the genomic content being manifested as elevated number of *oriC*s (Lee et al., 2003; Kadoya et al., 2011). Interestingly, *parAB* deletion may also lead to aberrations in the cell length (in *P. aeruginosa, M. smegmatis, C. glutamicum*) resulting from septum mispositioning or growth dysregulation (Donovan et al., 2013; Ginda et al., 2013). In some bacteria, *parAB* deletion results in more pleiotropic phenotypes, such as altered motility in *P. aeruginosa*, increased transformation competence in *S. pneumoniae*, reduced resistance to γ-radiation in *Deinococcus radiodurans*, and inhibited sporulation in *B. subtilis* (Errington, 2003; Lasocki et al., 2007; Bartosik et al., 2009; Charaka and Misra, 2012; Attaiech et al., 2015). Similarly, elimination of condensins has a bacterial species-dependent impact on chromosome organization. In *E. coli* and *B. subtilis*, the deletion of the genes encoding condensins results in a severe growth phenotype and chromosome segregation defects, while their deletion in other bacteria (*P. aeruginosa, M. smegmatis, S. coelicolor*) leads to a mild phenotype (reviewed by Nolivos and Sherratt, 2014). These observations reinforce the idea that segregation proteins are involved in multiple and varied cellular processes.

### Other Proteins Involved in Chromosome Segregation

Interestingly, not all bacterial species employ the ParA and ParB proteins to segregate chromosomes. While the coccoid *S. pneumoniae* possesses a ParB homolog, it lacks ParA. In these bacteria, ParB cooperates with SMC proteins in chromosome segregation (Minnen et al., 2011). Moreover, some γ-proteobacteria, including *E. coli*, do not possess any ParA or ParB homologs. In contrast to ParAB-driven segregation, in *E. coli*, the segregation of newly replicated *oriC* regions is delayed by their cohesion. Cohesion is controlled by TopoIV, a type II topoisomerase, and SeqA, a protein involved in the regulation of replication initiation (Lu et al., 1994; Joshi et al., 2013; Dewachter et al., 2018). Interestingly, in response to DNA damage-induced stress, the SMC homolog RecN contributes to cohesion control (Vickridge et al., 2017). Moreover, in *E. coli*, additional *ori* domain-organizing factors were shown to contribute to positioning of the *oriC* region. These factors include the *cis*-acting sites and *maoS* bound by the characteristic of *E. coli* MaoP protein as well as *migS* sites (Yamaichi and Niki, 2004; Valens et al., 2016; Dame et al., 2019). Finally, in *E. coli*, MukB, a structural SMC homolog strongly contributes to chromosome segregation (Hiraga et al., 1991; Yamazoe et al., 1999). Interestingly, in *E. coli*, in contrast to SMC in *C. crescentus* and *B. subtilis*, MukB does not cause chromosomal arms to adopt a longitudinal arrangement. MukB cooperates with TopoIV and the *ter* domain-organizing protein MatP (Nolivos et al., 2016; Lioy et al., 2018). MatP-dependent *ter* organization is a characteristic and unique feature of enterobacteria (Mercier et al., 2008). Whether there is any evolutionary advantage to abandoning the *parABS* system and adopting the another chromosome arrangement in enterobacteria has not yet been addressed. However, it is tempting to conclude that an elaborate life cycle and/or cell shape (e.g., *C. crescentus*) demand more complex chromosome segregation machinery.

As the last step of chromosome segregation, the separation of the duplicated chromosome *ter* regions is the final, critical checkpoint in this process. Interestingly, segregation of the *ter* regions was observed to be delayed in a number of bacterial species (Thiel et al., 2012). The segregation of *ter* regions requires the activity of accessory proteins, among which the chromosome translocase FtsK is the most widespread (Massey et al., 2006; Stouf et al., 2013; Crozat et al., 2014). The translocase family also includes the SpoIIIE protein, which is responsible for packaging of the chromosome into the small space of the forespore during *B. subtilis* sporulation, and TraB homologs involved in the conjugal transfer of DNA (Vogelmann et al., 2011; Thoma and Muth, 2015). As part of the divisiome, FtsK is associated with the newly formed septum via its N-terminal domain, while its C-terminal domain is involved in ATP hydrolysis-dependent DNA translocation as well as recombinase activation (Löwe et al., 2008; Grainge, 2013; Keller et al., 2016). FtsK activity is thus accompanied by DNA decatenation and recombination carried out by TopoIV and XerCD recombinase, respectively (El Sayyed et al., 2016). Replication and segregation of the *ter* region are tightly coordinated with Z-ring dynamics and the progression of cell division (Espéli et al., 2012; Adams et al., 2014).

Although the main players in the chromosome segregation have been identified, the mechanisms by which the activities of segregation proteins are regulated remain largely unexplored. Nevertheless, the emerging picture is that the process of the chromosome segregation is adjusted to the cell physiological state. Chromosome segregation may be regulated by modulation of the interaction between segregation proteins and DNA, nucleotide binding or other posttranslational protein modifications. Since the activities of various segregation proteins (ParA, SMC/MukB, FtsK, TopoIV) are dependent on the ATP hydrolysis, the overall energetic load of the cell, as manifested by its ATP level, should be considered as an important factor that modifies the efficiency of the segregation process. Importantly, the activities of the segregation proteins may be modulated due to their interactions with protein partners. Subsequently, these interacting partners may alter not only the efficiency of chromosome segregation but also other cell cycle parameters due to their engagement in cell division or cell elongation.

## The ParB Complex—Regulation of Its Formation and Its Impact on Chromosome Dynamics

**FIGURE 2:**
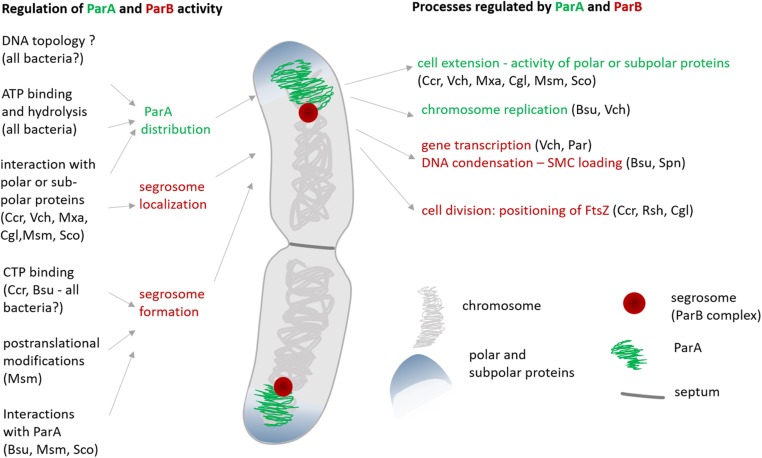
The regulation of ParA and ParB activity and the impact of these proteins on cellular processes other than *oriC* segregation. The abbreviation indicate the species in which particular interactions or influence on activity have been observed: Cgl, *C. glutamicum*; Cre, *C. crescentus*; Bsu, *B. subtilis*; Msm, *M. smegmatis*; Mxu, *M. xanthus*; Pae, *P. aeruginosa*; Rsh, *R. sphaeroides*; Sco, *S. coelicolor*; Spe, *S. pneumoniae*; Vch, *V. cholerae*; ParA-related regulation is shown in green, ParB-related regulation is shown in red, question mark indicates the connection that has been suggested but has not yet been experimentally confirmed.

To fulfill their functions, segregation proteins must interact with DNA; hence, the modification of their DNA affinity is critical for the regulation of their activity. Studies in various model bacteria have reported the modification of segrosome formation by the interaction of ParB with CTP or ParA (Breier and Grossman, 2007; Ginda et al., 2013; Baek et al., 2014; Donczew et al., 2016; Osorio-Valeriano et al., 2019; Soh et al., 2019) (Figure 2). Moreover, transcriptional regulation and posttranscriptional modifications of segregation proteins have been described.

### Transcriptional Regulation of Segregation Genes

Formation of protein complexes may be controlled by transcriptional regulation. In *C. crescentus*, this mode of regulation applies to primary cell cycle coordinators, such as the replication initiator DnaA and the cell division protein FtsZ (Laub et al., 2000; Frandi and Collier, 2019). The transcriptional regulation exerted by master regulators such as CtrA and GcrA allows the functional differentiation of two daughter cells, which is characteristic of *C. crescentus* (Kirkpatrick and Viollier, 2012; Tsokos and Laub, 2012). While the stalked cell is capable of undertaking a new round of chromosome replication, in the swarmer cell, chromosome replication and cell division are inhibited. In *C. crescentus*, the transcription of genes encoding proteins involved in chromosome topology maintenance (topoisomerases and NAPs) was shown to be developmentally controlled, but the cell cycle-dependent transcriptional regulation of genes encoding segregation proteins has not been reported (Holtzendorff et al., 2004). Fluctuations in ParA and ParB levels between cell divisions cannot be excluded; however, there is no evidence of such regulatory mechanisms in any studied unicellular bacterium. Interestingly, the autoregulation of *parAB* was demonstrated in case of plasmid segregation proteins (Kwong et al., 2001). The only report showing the life-cycle associated transcriptional induction of chromosomal *parAB* genes comes from *S. coelicolor*, a mycelial bacteria that undergo sporulation in response to stress, particularly nutrients limitation. *Streptomyces* sporulation involves the conversion of multigenomic sporogenic hyphae into chains of unigenomic spores (Flärdh and Buttner, 2009). While during mycelial vegetative growth, cell divisions are rare and not accompanied by chromosome segregation, sporulation requires the synchronous segregation of multiple chromosomal copies synchronized with multiple cell divisions (Jakimowicz and van Wezel, 2012). In *S. coelicolor* and *S. venezuelae, parAB* genes, similarly as *ftsZ*, are controlled by cell cycle regulators associated with the onset of sporulation (WhiA and WhiB) (Flärdh et al., 2000; Jakimowicz et al., 2006; Bush et al., 2013, 2016). Their upregulation allows the induction of *parAB* and *ftsZ* required for the formation of numerous ParB complexes and Z-rings, respectively, along sporogenic hyphal cells (Schwedock et al., 1997; Kim et al., 2000; Jakimowicz et al., 2005, 2007). The unprecedented transcriptional upregulation of *parAB* in *Streptomyces* fulfils the demands of their unique cell cycle and unusual mode of the chromosome segregation.

### The Regulatory Role of Nucleotide Binding and Posttranslational Modifications in ParB

Little is known about the regulation of segrosome architecture; however, CTP binding by ParB was recently demonstrated to modulate interactions during ParB complex formation (Osorio-Valeriano et al., 2019; Soh et al., 2019). CTP is specifically bound within the ParB box II motif, which was previously shown to contribute to the long-range interactions (Graham et al., 2014). *ParS* binding by ParB induces CTP hydrolysis and increases the protein affinity toward the *parS* sequence, presumably leading to the complex rearrangement (Osorio-Valeriano et al., 2019; Soh et al., 2019). While the binding of CTP to ParB was demonstrated in *B. subtilis* and *M. xanthus*, the CTP interaction interface is highly conserved among ParB homologs, suggesting that this feature is preserved. Moreover, CTP binding by plasmid-encoded ParB proteins (F plasmid ParB_F_ and P1 prophage ParB_P1_) was demonstrated reinforcing the significance of nucleotide binding for complex formation (Soh et al., 2019). Since CTP is primarily involved in biosynthesis of nucleic acids and phospholipids, rather than the storage and transfer of energy, its intracellular level may reflect the cell capacity to replicate its DNA. Moreover, CTP biosynthesis is tightly regulated, and its level changes in a growth phase-dependent manner (Meng and Switzer, 2001; Jørgensen et al., 2003; Walker et al., 2004). Interestingly, in *E. coli*, one of NAP (Fis) was shown to be transcriptionally controlled by CTP, indicating the impact of this nucleotide on chromosome topology (Walker et al., 2004). Thus, the dependence of segrosome formation on subcellular CTP levels may influence functional segregation complex formation under unfavorable conditions, such as nutrients limitation, and serve as a link between cell physiology and chromosome segregation.

Furthermore, circumstantial evidence suggests that factors dependent on the cell physiological state, other than nucleotide levels, may also affect segrosome formation. Posttranslational modifications, especially phosphorylation, are well described mechanism to fine-tune the activity of the various proteins in response to changes of environmental conditions, e.g., nutrients availability (Stock et al., 1989; Bernal et al., 2014; Carabetta and Cristea, 2017; Janczarek et al., 2018). While number of nucleoid associated proteins (NAPs) including HU-like proteins were shown to be phosphorylated in *B. subtilis* and *M. tuberculosis* and plethora of DNA organizing proteins were suggested to be target for phosphorylation, the evidence for phosphorylation of chromosome segregation proteins is limited (Gupta et al., 2014; Garcia-Garcia et al., 2016). In mycobacteria, ParB was reported to be influenced by phosphorylation, which modified protein affinity toward DNA and abolished its interaction with ParA (Baronian et al., 2015). ParB was shown to be phosphorylated *in vitro* by several eukaryotic-like Ser/Thr protein kinases whose main role is cell adaptation to changing environmental conditions (e.g., nutrient accessibility). Phosphorylation may potentially mediate fluctuations in ParB activity in relation to cell conditions; however, there is no experimental evidence of such a regulatory mechanism. Among the other posttranslational modifications, acetylation was also shown to influence the activity of numerous DNA associated proteins including topoisomerase I (in *E. coli*), DNA repair protein Ku and NAP HU (in *M. smegmatis*) (Zhou et al., 2015, 2017; Ghosh et al., 2016; Anand et al., 2017; Carabetta and Cristea, 2017), however, there are no reports of acetylation influencing directly the activity of chromosome segregation proteins ParA or ParB.

### The Influence of ParA on Segresome Assembly

In some bacterial species assembly of the ParB complex was shown to be influenced by ParA. ChIP analyses of *B. subtilis*, *V. cholerae*, *S. venezuelae*, and *M. smegmatis* indicated that elimination of ParA decreased ParB binding to at least some *parS* sites (Breier and Grossman, 2007; Ginda et al., 2013; Baek et al., 2014; Donczew et al., 2016). Additionally, in *P. aeruginosa*, the impact of the ParB complex on chromosome structure was shown to be dependent on ParA (Bartosik et al., 2014). This could be explained by the lower level of ParB in *parA* deletion strains (detected in *B. subtilis* and *P. aeruginosa* but not in *S. venezuelae* and *M. smegmatis*) but may also suggest that ParA promotes ParB complex rearrangement. The latter idea is reinforced by the observed influence of ParA on ParB binding to DNA *in vitro* (Jakimowicz et al., 2007). Because ATP hydrolysis is critical for the activity of ParA, changes in intracellular ATP levels, which reflect fluctuations in the cell energetic state, may impact segrosome formation. In fact, exposure of *M. smegmatis* cells to stressful conditions modified ParA localization (Ginda et al., 2013; Pióro et al., 2019). Finally, it should be considered that environmental conditions such as increased temperature, pH or osmotic stress influence the chromosome topology (Dorman and Dorman, 2016; Qin et al., 2019), and changes of chromosome topology affect the binding of numerous DNA-interacting proteins including SMC and NAPs (Gruber and Errington, 2009; Dorman and Dorman, 2016; Tran et al., 2017; Qin et al., 2019). Consequently, the activities of segregation proteins may also be easily modified by changes in chromosome topology induced by environmental stress (Figure 2).

### Segrosome Impact on Chromosome Topology and Gene Expression

While segrosome assembly may be adjusted in response to environmental clues, its architecture has a profound impact on chromosome structure. In *S. coelicolor*, segrosomes recruit topoisomerase I, which is required to resolve topological problems and proceed with chromosome segregation (Szafran et al., 2013). Alteration of the ParB complex architecture by changing the *parS* site position was shown to diminish *C. crescentus* fitness (Tran et al., 2018). Interestingly, in *B. subtilis* and *S. pneumoniae*, changes in the positions of *parS* sites resulted in the redistribution of ParB but had little effect on chromosome segregation and culture growth (Broedersz et al., 2014; Attaiech et al., 2015; Wang et al., 2015). However, the function of the segrosome in recruiting SMC proteins was affected by abolished ParB bridging (Gruber and Errington, 2009; Minnen et al., 2011; Graham et al., 2014; Wilhelm et al., 2015). Thus, the abovementioned studies indicate that changes in segrosome architecture influence the efficiency of chromosome compaction.

The large nucleoprotein complex formed by ParB is bound to influence chromosome topology and consequently gene expression (Figure 2). The first observation of segregation complex influence on gene expression was made for plasmid ParB proteins (Lynch and Wang, 1995; Rodionov et al., 1999). Considering the interspecies differences in segrosome organization, the impact of this complex on chromosome topology may be diverse. Indeed, in *S. pneumoniae*, formation of the ParB complex affects the activities of only adjacent genes, particularly the *com* operon (located 5 kb from *parS* sites), which encodes proteins involved in competence. This observation explains the increased competence of a *S. pneumoniae parB* deletion strain. Similar to *S. pneumoniae*, in *V. cholerae*, the binding of ParBI to 3 *parS* sites, results in limited ParB spreading and affects the transcription of only some of the genes in the region bound by ParB (3 of 20 genes) (Baek et al., 2014). Moreover, the transcription of several genes outside of the region bound by ParB is modified in the *parAB* deletion strain. In contrast to *S. pneumoniae* and *V. cholerae*, in *P. aeruginosa*, ParA and ParB elimination and their overexpression has been shown to affect transcription globally, influencing the expression of genes encoding stress response proteins and putative transcriptional regulators (Bartosik et al., 2014). This phenomenon was explained by *P. aeruginosa* ParB binding non-restricted to consensus *parS* sites and ability of this protein to interact with short *parS*-like motifs (Kawalek et al., 2018). This low DNA-binding specificity of ParB suggests its role in the general organization of DNA, similar to the role of NAPs. Surprisingly, in contrast to the abovementioned bacteria, in *B. subtilis*, the influence of the ParB complex on gene expression could not be detected (Breier and Grossman, 2007). Although preliminary studies suggested the involvement of *parAB* in the regulation of sporulation, this phenomenon was later shown to be independent of transcriptional regulation but was explained by the regulation of DnaA activity by ParA (see below). However, other studies reported that deletion of *parAB* in *B. subtilis* activated the SOS response by inducing a *recA* and the gene encoding the cell division inhibitor YneA (Bohorquez et al., 2018). Thus, the influence of the segrosome on the transcription of at least some genes is a common feature of the ParB complex.

## Coordination of the Cell Cycle—The Role of Segregation Protein Interactions

Segregation proteins interact with not only DNA and each other but also with proteins engaged in the key cell cycle processes. The ParA–DnaA interaction links chromosome segregation with chromosome replication, the interactions of ParA with polar proteins impact cell elongation, and the ParB–MipZ interaction controls the cell division (Marczynski et al., 2019) (Figure 2). ParA and ParB interaction partners may contribute to the chromosome segregation process; on the other hand, their activity may be controlled by ParA and/or ParB. Importantly, the DNA binding of segregation proteins modulates their availability to the partner-proteins interactions (Murray and Errington, 2008; Schofield et al., 2010; Pióro et al., 2019). Interestingly, most interactions with ParA and ParB are specific to bacterial genera, although some are more widespread and have been detected in various bacterial species.

### Interactions Between Segregation and Polar or Subpolar Proteins

The interactions between segregation proteins and polar and subpolar proteins result in the specific localization of *oriC*s in *C. crescentus, V. cholerae, M. xanthus, M. smegmatis*, and *S. coelicolor*, all of which anchor the *oriC* region at their poles or subapically (Bowman et al., 2008; Ebersbach et al., 2008; Yamaichi et al., 2012; Ginda et al., 2013; Kois-Ostrowska et al., 2016; Lin et al., 2017; Pióro et al., 2019). During the asymmetric cell division of *C. crescentus*, the unidirectional chromosome segregation must be precisely controlled. The interaction between the ParB protein and the polarity factor PopZ positions the *oriC* region at the old pole before chromosome replication. PopZ is a small acidic protein that oligomerizes to form a mesh-like structure. In addition to its role in the *oriC* anchoring, its primary role is to recruit factors involved in stalk morphogenesis (Bowman et al., 2008; Ebersbach et al., 2008). Soon after the initiation of chromosome replication, one of the duplicated segrosomes is moved from the old cell pole to the new pole, and PopZ is simultaneously redistributed to form bipolar foci (Bowman et al., 2008; Ebersbach et al., 2008). Interestingly, PopZ also interacts with ParA monomers released from DNA upon ParA interaction with the ParB complex. Thus, PopZ was suggested to be involved in nucleotide exchange and the regeneration of ParA-ATP bound dimer and restoring its DNA-binding activity. Interestingly, the role of PopZ in regulating ParA activity is partially synergistic with the function of another *C. crescentus* ParA interaction partner – the coiled-coil TipN protein. TipN is mainly localized at the new pole, and the ParA–TipN interaction is critical for the ParA distribution and the directionality of segrosome movement (Lam et al., 2006; Ptacin et al., 2010). Importantly, ParA was shown to influence the function of PopZ; accumulating at the new pole ParA recruits PopZ, generating a nucleation site that initiates PopZ polymerization (Laloux and Jacobs-Wagner, 2013). It should be noted that formation of PopZ–ParA complex is dependent on availability of ParA released from nucleoid, most often by ongoing chromosome segregation (Laloux and Jacobs-Wagner, 2013). Since PopZ recruits the cell cycle regulator CtrA and its associated kinase as well as CtrA-targeting protease ClpXP (Joshi et al., 2018), the ParA control of PopZ localization possibly indicates the coordination of chromosome segregation with the global cell cycle regulation.

Similar as in *C. crescentus*, polar anchoring of *oriC* region and unidirectional chromosome segregation was described for *V. cholerae* chromosome I (the larger of the two *V. cholerae* chromosomes). In *V. cholerae*, ParAI (the ParA protein that governs the segregation of chromosome I) interacts with the polar localized protein HubP, and deletion of *hubP* abolished polarly *oriCI* positioning (Yamaichi et al., 2012). HubP also interacts with chemotactic machinery and flagellar proteins; moreover, HubP was also identified to interact with two other ATPases, ParC and FlhG (Yamaichi et al., 2012). Interestingly, in *Shewanella oneidensis* HubP homolog was also shown to be involved in chromosome segregation. Moreover, the identified in these bacteria interaction between HubP and PdeB, phosphodiesterase that controls c-di-GMP level in the cell, may indicate the potential link between cyclic nucleotide signaling and chromosome segregation (Rossmann et al., 2019). An interesting example of a bacterium in which the *oriC* is not localized at the poles but rather exhibits subpolar localization is *M. xanthus.* In this bacterium, the positioning of *oriC* is exerted by the ParA interaction with PadC, which in turn binds the bactofilin scaffold stretching from the poles (Lin et al., 2017). Only the monomeric form of ParA is recruited to the bactofilin–PadC complex, which is reminiscent of the ParA–PopZ interaction in *C. crescentus* (Lin et al., 2017). However, in case of *V. cholerae* and *M. xanthus*, there is no evidence that ParA influences the activity or localization of its interaction partners.

In the apically extending cells of actinobacteria segregation proteins also interact with polar protein complexes. The main component of the polar complex in these bacteria is the coiled-coil tropomyosin-like protein DivIVA, which recruits peptidoglycan-synthesizing machinery to the poles (Kang et al., 2008; Letek et al., 2008; Flärdh, 2010; Hammond et al., 2019). In *M. smegmatis*, DivIVA directly interacts with ParA (Ginda et al., 2013). The inhibition of this interaction was shown not only to decrease the efficiency of the chromosome segregation, but also it visibly increased the cell elongation rate, indicating ParA influence on DivIVA activity (Pióro et al., 2019). Considering that the recruitment of ParA to DivIVA was proved to compete with ParA–DNA interaction, the release of ParA from DNA upon interaction with ParB complex or, conceivably, due to changes of DNA topology, may have the impact on cell elongation rate. Markedly, DivIVA in mycobacteria is phosphorylated by the PknA kinase, the activity of which is regulated by the PknB kinase, and both PknA and PknB are essential Ser/Thr protein kinases that control growth rate and morphology (Kang et al., 2005; Jani et al., 2010; Lee et al., 2014). In response to the extracellular signals, these kinases phosphorylate regulators of central carbon metabolism and proteins involved in the stress response, transport and cell wall synthesis. It was shown that growth phase dependent DivIVA phosphorylation status regulates the rate of peptidoglycan synthesis (Jani et al., 2010). Whether the phosphorylation status of DivIVA influences its interaction with ParA, linking environmental conditions with the segregation of chromosomes, remains to be elucidated.

Unlike *M. smegmatis*, in *S. coelicolor*, which also belongs to actinobacteria, the interaction between ParA and DivIVA was not detected, but ParA was found to directly interact with the Scy protein – the other component of a polar protein complex named the polarisome, which also includes DivIVA. During *S. coelicolor* vegetative growth, this interaction is responsible for anchoring of the apical chromosome at the tips of multigenomic hyphal cells. Importantly, the deletion of *parA* affected the rate of hyphal growth, which was explained by ParA-dependent modulation of Scy activity (Ditkowski et al., 2013; Donczew et al., 2016). Developmentally controlled ParA accumulation during sporulation leads to polarisome disassembly and inhibits hyphal elongation (Ditkowski et al., 2013). Interestingly in *S. coelicolor* ParA was shown to interact with the other segregation protein ParJ, however, the contribution of this protein to the segregation process is not fully understood (Ditkowski et al., 2010). Unlike in above described actinobacteria, in closely related *C. glutamicum* ParB directly interacts with DivIVA and this interaction positions *oriC* at the cell pole. The observation that deletion of *parB* results in impaired cell extension indicates that ParB–DivIVA interaction may impact DivIVA activity (Donovan et al., 2010, 2012). Thus, in actinobacteria, the interactions of segregation proteins with polar complexes not only contribute to chromosome segregation but also regulate cell elongation.

Interestingly, DivIVA is also involved in anchoring the *oriC* region during *B. subtilis* sporulation, though not through its direct interaction with ParAB homologs; DivIVA instead interacts with a complex containing MinD and MinJ (Kloosterman et al., 2016). Moreover, the additional RacA protein, which also specifically binds the *oriC*-proximal part of the chromosome, contributes to *oriC* anchoring in *B. subtilis* (Ben-Yehuda et al., 2005; Schumacher et al., 2016).

### Interactions Between Segregation and Replication Proteins

In a number of bacterial species, ParA was also shown to be involved in the regulation of the chromosome replication. The direct interaction of ParA with DnaA was first described in *B. subtilis*, in which ParA and ParB homologs were originally identified as regulators of sporulation and called Soj and Spo0J, respectively. The elimination of Spo0J was found to inhibit sporulation and that inhibition may be counteracted by deletion of the gene encoding Soj (suppressor of Spo0J, a homolog of ParA) (Quisel et al., 1999). Later studies showed that this effect is indirect and results from Soj-dependent regulation of DnaA, which subsequently negatively regulates transcription of sporulation genes (Murray and Errington, 2008; Scholefield et al., 2011, 2012). Monomeric Soj directly interacts with DnaA and reduce its interaction with DNA inhibiting its oligomerization. In the absence of Spo0J, the DNA-bound Soj dimer is more stable, and the level of monomeric Soj available to interact with DnaA is decreased; therefore, DnaA replication activity is elevated. Thus, the function of Soj as a DnaA inhibitor depends on interaction between segregation protein and DNA. Since DNA binding by ParA homologs may be influenced by intracellular ATP level, Soj likely links the changes of the cell physiological state and environmental conditions that have the impact on cell energetic state with the DnaA replication (Murray and Errington, 2008; Scholefield et al., 2011, 2012). Additionally, in *V. cholerae*, DnaA interacts with ParA as well as ParB, while in *D. radiodurans* (another bacterium with a multipartite genome: two chromosomes and a megaplasmid), the DnaA protein interacts with ParB (Kadoya et al., 2011; Maurya et al., 2019). The involvement of the segregation proteins in replication regulation explains the increased number of *oriC* resulting from *parB* deletion.

### Interactions Between Segregation and Cell Division Proteins

Chromosome segregation proteins also interact with the cell division proteins. In the α-proteobacteria *C. crescentus* and *R. sphaeroides*, the interaction between the ParB protein and MipZ (a ParA homolog) was detected (Thanbichler and Shapiro, 2006; Dubarry et al., 2019). MipZ is an inhibitor of FtsZ polymerization that exhibits dynamic localization characteristic of the ParA family of ATPases. In *C. crescentus*, MipZ forms a cloud-like structure with the lowest MipZ concentration in the middle of the cell, which restricts Z-ring formation to the cell center (Thanbichler and Shapiro, 2006). The localization of MipZ in *R. sphaeroides* is different; MipZ is situated mainly at the cell poles but also at the mid-cell position. In both *C. crescentus* and *R. sphaeroides*, the localization of MipZ depends on ParB, but unlike ParA, MipZ dimers are recruited and stabilized by ParB (Thanbichler and Shapiro, 2006; Dubarry et al., 2019). Importantly, in *C. crescentus*, the transcription level of *mipZ* changes during cell cycle progression and in response to environmental cues (e.g., nitrogen starvation) (Collier, 2019). In *C. glutamicum*, ParB also interacts with PldP – a ParA homolog involved in the regulation of cell division (Donovan et al., 2010). Moreover, in these bacteria, the direct interaction between ParB and FtsZ—a cell division initiator—was shown (Donovan et al., 2010). These interactions presumably account for the observed influence of *parAB* deletion on septum placement (Donovan and Bramkamp, 2014).

Interestingly, in *S. pneumoniae* (which lacks the ParA component of the *parABS* system), ParB interacts with CpsD, which is homologous to ParA tyrosine (BY) kinase and localizes at the site of cell division (Bender and Yother, 2001). BY-kinases are autokinases that regulate polymerization and the export of capsular polysaccharides. Inhibition of CpsD phosphorylation delayed chromosome segregation, while increased CpsD phosphorylation enhanced ParB mobility. The interaction between ParB and CpsD may coordinate chromosome segregation with capsular formation and the cell division (Nourikyan et al., 2015). Recent studies identified another ParB interacting partner in *S. pneumoniae*—the RocS protein. RocS is required for chromosome segregation but also interacts with FtsZ and CspD (Mercy et al., 2019). The above examples show that chromosome segregation and cell division are coupled due to the interactions of segregation proteins.

Some of the interactions of segregation proteins were shown to be critical under stress conditions. These include discovered in *M. smegmatis* interaction between ParA and 3-methyladenine DNA glycosylase, a protein mainly involved in DNA repair (Huang and He, 2012). This interaction stimulates the ATPase activity of ParA and regulates cell growth and morphology independent of DNA glycosylase activity. In *B. subtilis*, ParAB was shown to cooperate with another segregation protein, WhiA, which was suggested to maintain DNA integrity (Bohorquez et al., 2018). Interestingly, double *parAB*/*whiA* deletion was lethal and could be explained by the blockade of cell division.

## Concluding Remarks

Studies of last two decades have shed light on chromosome segregation, revealing the concerted actions of segregation proteins, dissecting the mechanisms of their activities and describing their interactions. However, evidence that the chromosome segregation process is adjusted to environmental conditions has only started to emerge. Environmental stress factors, such as nutrients limitation, modify cell physiology and require adjustment of the cell cycle process. The possible pathways that can be used to coordinate the cell cycle with stress response are those based on intracellular nucleotide levels and chromosome topology. In particular, the finding that the ParB–CTP interaction is a critical factor for segrosome formation opens a new avenue for the exploration of chromosome segregation regulation. Finally, the impact of polar proteins (TipN, PopZ, HubP, or DivIVA) on the activities of segregation proteins under unfavorable conditions also remains to be further investigated to identify the links between changes in cell physiology and chromosome segregation.

Furthermore, recent studies have indicated the impact of chromosome segregation proteins on other cell cycle processes. Interestingly, due to their involvement in highly species-specific interactions (including both DNA interactions during segrosome formation and protein–protein interactions), the involvement of segregation proteins in coordination of the cell cycle is diverse and species dependent. Common regulatory pathways (identified in at least two unrelated organisms) include the regulation of gene transcription, chromosome replication, and the regulation of the cell elongation and division (Marczynski et al., 2019). Notably, the availability of ParA to interact with their protein partners (DnaA, PopZ, DivIVA) depend on the ParA binding to chromosome. Since this interaction is plausibly influenced by environmental factors, it may serve as the important regulatory circuit. However, further studies are required to fully understand the complex regulatory networks behind the identified connections and the impact of external factors on the global coordination of cell cycle processes.

## Author Contributions

MP and DJ wrote the manuscript.

## Conflict of Interest

The authors declare that the research was conducted in the absence of any commercial or financial relationships that could be construed as a potential conflict of interest.
